# Some Resources in the Diagnosis of Nervous Diseases
*The substance of a paper read before the Harveian Society, May 13th, 1897, corrected by the author.


**Published:** 1897-05-22

**Authors:** James Cagney

**Affiliations:** Physician in charge of Electrical Department, St. Mary's Hospital; Physician, Hospital for Epilepsy and Paralysis; Assistant Physician, North-West London Hospital


### SOME RESOURCES IN THE DIAGNOSIS OF NERVOUS DISEASES.*

By James Cagney, M.A., M.D., M.R.C.P., Physician in charge of Electrical Department, St. Mary's Hospital; Physician, Hospital for Epilepsy and Paralysis; Assistant Physician, North-West London Hospital.

There are certain matters touching the diagnosis of diseases of the peripheral portion of the nervous system which, although no longer novel, are not yet so commonly known and daily practised in the profession as they ought to be. It must be distinctly understood that in speaking of the peripheral nervous system one means thereby the entire tract, beginning with and including the motor cells in the anterior columns of the spinal cord and medulla, and extending to and including the muscular fibres whose function and nutrition are dependent upon them. A sufficient lesion of any kind, implicating any part of this tract, will effect a definite modification of the electrical signs, a modification always the same, or differing only in degree, whatever the nature of the lesion, or in whatever part of the tract it may be placed. On the other hand, no lesion, however severe, of any part of the spinal cord, above or outside this tract, as, for instance, a lesion of the posterior columns such as belongs to locomotor ataxy, or of the lateral columns as in spastic paraplegia, and no lesion whatever of any part of the brain, will cause a similar change in the electrical reactions.

This limitation of the morbid effects to the lower regions of the motor tract constitutes the chief value of the electrical tests, because generally speaking when a question arises as to the nature or the cause of a paralysis, the only ambiguity is as to whether this lower region is involved or not.

Paralysis is a symptom common to all lesions of any part of the motor tract, and to some conditions, of which hysteria is one, in which no lesion of the motor tract can be recognised by our present means. In the presence, then, of paralysis, it becomes a matter of the greatest importance to ascertain whether or no the lower region is involved, for this is the point with which diagnosis, prognosis, and treatment are directly concerned.

It being premised that the electrical changes which are now in question belong only to peripheral nervous degenerations—to degeneration of the neuron, and possibly to rare and idiopathic muscular changes—I shall speak of the condition, whatever its clinical character, as peripheral neuritis; but it must be recognised that I do this only for the sake of brevity, and because the term is commonly employed in medicine. There can be little doubt that the clinical states included under the term are destined, when further knowledge is available, to be quite separated from inflammatory changes.

Of all diseases of the nervous system the degeneration of the peripheral nerves which we call "peripheral neuritis" is the most definite, certain, and recognisable entity, for of it we have a physical sign of absolute and unerring certainty, one obtainable at will, and one which indicates degenerative changes in the nerve fibre as plainly as if it had been stained and mounted and submitted to examination under the microscope.

The disease is a common one, and although it is a formidable malady yet it is infinitely more amenable to treatment than, for example, is Bright's disease. Surely, then, it is incumbent upon medical men to be provided with the means of arriving at a diagnosis which is so absolute and certain. No medical man would be excused overlooking the presence of albuminuria. Why, then, should he not feel bound to be as well versed in the electrical tests for degenerating nerve and muscle as he is for the chemical tests for albumen in the urine?

The reaction of degeneration is the term used to describe the behaviour under electrical stimulation of a muscle—meaning by the term a considerable group of muscular fibres—when the motor nerve of the latter is undergoing destruction or repair. Under such circumstances this reaction is never wanting, and moreover the pathological condition to which it testifies is always esentially the same. Wherever in the course of the neuron the morbid change begins there will be from that point downwards a definite change in the neuro-muscular elements, as a consequence of which their reaction to the two forms of electrical current will be altered.

In the application of electricity for the purpose of diagnosis what is essential is—(1) That we should be able to bring the nerve or muscle to be examined under the influence of the current; and (2) in regard to the constant current that we should be quite certain of submitting the nerve to the separate action of the positive or the negative pole as we wish it, for with the constant current the effects produced are polar, and differ characteristically with the pole used.

To give precision to the pole used locally a small electrode is used, while the other, the indifferent electrode, is a very large one, and is placed at a point anatomically as far as possible from the part under investigation—generally on the episternal or epigastric region.

When this arrangement is made the path of the current may be roughly represented by a cone, the density of the current increasing towards the apex; that is, towards the small electrode. The nearer, then, the nerve lies to the small electrode, and the larger and more distant the other, the greater will be the likelihood that the nerve is submitted to the unmixed influence of the pole used locally. Let us get rid of all notions about ascending and descending currents, and think only of the pole which we apply to the point examined.

In health, if a faradic current be applied by any pole to any part of a motor nerve or muscle, a tetanic contraction results. In degeneration a faradic current, wherever and however applied, will fail to give a contraction. Thus the entire absence of faradic contraction in a muscle is a certain sign that there is a lesion somewhere in the neuro-muscular tract—that is, somewhere not higher than the group of cells in the anterior columns of the spinal cord.

It must be remembered, however, that the presence even of a powerful contraction under faradism does not exclude such a lesion. Healthy fibres and others undergoing degeneration may lie side by side in the

* The substance of a paper read before the Harveian Society, May 13th, 1897, corrected by the author.

same muscle; the contraction of the first may mask the inactivity of the latter, and in such a case a negative test may prove nothing. Again, the absence of a faradic contraction may mean, besides degeneration, the entire destruction of and absence of nerve or muscle substance, and for this reason also it is indispensable to have a test which will distinguish the two conditions. Such a test is afforded by the constant current.

Given a healthy nerve and muscle, the stimulation of either with either pole of the current gives a quick, lightning-like contraction whenever the current is made or broken, and this contraction occurs with a weaker current when the negative pole is used than when the positive pole is used. If, however, the nerve has been injured, and is undergoing degeneration or repair, one can obtain no response to any stimulation of the nerve.

To obtain any result the electrode must be placed directly over the muscle, and when this is done it is found that the positive and not the negative is the more efficient stimulus. Again—and this is a most significant difference—the contraction is no longer quick and lightning-like, but is slow, long drawn, and exhibits a remarkable vermicular progress.

If in a muscle there are both healthy and diseased fibres the practised eye can distinguish the two sorts of contraction; and the slow vermicular movement, even in a few fibres, is absolutely diagnostic.

It is well to point out again that this reaction can only take place in motor nerve fibres, and that the morbid changes which occur in sensory nerves, giving rise to neuralgias and other forms of perverted sensation, might escape demonstration by this means. But it must be remembered that most peripheral nerves are of mixed function, and that, as the most limited display of the reaction of degeneration in the associated motor fibres is conclusive, the test is almost invariably applicable.

We thus have a test of a most positive nature in regard to a condition which undoubtedly is the cause of many ailments which are at present ill understood, a test which can be applied with the greatest facility and with the greatest certainty, and one which I am sure is far less made use of by the profession than it ought to be.

